# The evaluation of facial features as markers of cardiometabolic health in women of post-reproductive age

**DOI:** 10.1093/emph/eoag014

**Published:** 2026-07-02

**Authors:** Weronika M Obrochta, Urszula M Marcinkowska, Matthew E Ball, Rebecca S A Brittain, Andrzej Galbarczyk, Magdalena Klimek, Ilona Nenko, Ian D Stephen, Sonja Windhager, Grazyna Jasienska

**Affiliations:** Department of Environmental Health, Faculty of Health Sciences, Jagiellonian University Medical College, Krakow, Poland; Jagiellonian University Medical College, Doctoral School of Medical and Health Sciences, Krakow, Poland; Department of Environmental Health, Faculty of Health Sciences, Jagiellonian University Medical College, Krakow, Poland; School of Psychology, Faculty of Media, Science and Technology, Bournemouth University, Bournemouth, UK; Department of Environmental Health, Faculty of Health Sciences, Jagiellonian University Medical College, Krakow, Poland; Department of Anthropology, Yale University, New Haven, CT, USA; Department of Environmental Health, Faculty of Health Sciences, Jagiellonian University Medical College, Krakow, Poland; Department of Human Behavior, Ecology and Culture, Max Planck Institute for Evolutionary Anthropology, Leipzig, Germany; Department of Environmental Health, Faculty of Health Sciences, Jagiellonian University Medical College, Krakow, Poland; Department of Environmental Health, Faculty of Health Sciences, Jagiellonian University Medical College, Krakow, Poland; School of Psychology, Faculty of Media, Science and Technology, Bournemouth University, Bournemouth, UK; Department of Evolutionary Anthropology, University of Vienna, Vienna, Austria; Human Evolution and Archaeological Sciences, University of Vienna, Vienna, Austria; Department of Environmental Health, Faculty of Health Sciences, Jagiellonian University Medical College, Krakow, Poland

**Keywords:** cardiometabolic diseases, post-reproductive women, facial traits

## Abstract

**Background:**

The early-life environment has a significant influence on health in later life, including the risk of cardiometabolic diseases (CMDs). Several biomarkers have been proposed as putative indicators of early-life environmental quality, including facial fluctuating asymmetry (FA) and facial averageness (AVE). This study aimed to explore the association between facial FA and AVE with CMDs risk factors in women of post-reproductive age.

**Methods:**

The participants were 248 post-reproductive women (M = 61.6, SD = 10.70) from the Mogielica Human Ecology Study Site in Poland. Standardized facial photographs were taken, and FA and AVE were calculated using geometric morphometrics. Fasting blood samples were collected, and blood pressure was measured directly. Body composition was assessed via bioelectrical impedance analysis. CMDs risk factors included blood pressure, total cholesterol, high-density and low-density lipoproteins, triglycerides, and glucose levels. Additionally, the risk of metabolic syndrome was determined for each participant. Cardiovascular disease risk was also evaluated using the Systematic Coronary Risk Estimation scale (SCORE). FA and AVE were modelled separately against each CMDs risk factor using hierarchical linear and logistic models.

**Results:**

No statistically significant associations were found between either FA or AVE and individual CMDs risk factors. Similarly, no relationships were observed between facial measurements and either SCORE or metabolic syndrome risk. Follow-up Bayesian analyses provided evidence that FA and AVE did not predict CMDs risk factors.

**Conclusions and implications:**

While facial asymmetry and averageness may influence perceptions of health, they are unlikely to serve as biomarkers of CMDs risk in older women.

## INTRODUCTION

### Cardiometabolic diseases

Early-life conditions shape physiology and metabolism, suggesting that the prenatal environment may influence adult risk of cardiometabolic diseases (CMDs) [1, 2]. The concept of CMDs represents a unified pathophysiological framework underlying the development of both metabolic dysfunctions and cardiovascular diseases (CVD) [3]. The term CMDs encompasses conditions such as cardiovascular disease, hypertension, type 2 diabetes, and metabolic syndrome (MS) [4]. CMDs are a global burden on human health, affecting ~830 million people worldwide in 2022, according to the World Health Organization. In 2021, diabetes directly caused 1.6 million deaths, with 47% occurring in patients under the age of 70 [5]. From 1990 to 2022, the prevalence of diabetes, adjusted for age, increased for women in 131 countries [5]. Another risk factor for CMDs is lipid levels, specifically cholesterol and triglycerides. Dyslipidaemia and hyperglycaemia are modifiable risk factors for CMDs [6]; hence, identifying individuals with metabolic abnormalities could allow for early intervention and the prevention of CMDs.

Postmenopausal women are a group with potentially increased metabolic abnormalities and a higher risk of CMDs compared with women of reproductive age [7]. This elevated risk stems from hormonal shifts—lower oestrogen and/or higher androgens—affecting glucose, lipid, and insulin metabolism [8]. Additionally, one of the defining features of menopause is a shift in adipose tissue distribution towards visceral compartments [9]. As a result, postmenopausal women have a higher risk of developing MS, hypertension, CVD, insulin resistance, type 2 diabetes, and metabolic dysfunction compared with premenopausal women [10].

Heightened lipid profile levels, which include total cholesterol (TC), low-density lipoprotein cholesterol (LDL), and triglycerides, increase the risk of developing CMDs. Abnormal lipid levels (dyslipidaemia, TG ≥1.7 mmol/l and high-density lipoprotein (HDL)-cholesterol <1.3 mmol/l for women), heightened fasting blood glucose (dysglycaemia, >5.6 mmol/l), systolic blood pressure (SBP) (≥140 mmHg), diastolic blood pressure (DBP) (≥90 mmHg), and pulse pressure (difference between SBP and DBP, ≥60 mmHg) are also associated with increased risk of cardiovascular disease [11].

One possible factor related to the risk of developing CMDs is an adverse prenatal environment. According to the Developmental Origins of Health and Disease framework, prenatal environmental conditions can induce long-term changes in physiological systems, thereby shaping health outcomes in later life [12]. Low birth weight is a key marker of adverse prenatal conditions, including inadequate maternal nutrition or elevated prenatal stress exposure. It is consistently associated with increased risk of hypertension, type 2 diabetes, and cardiovascular disease in adulthood [2]. Among the proposed mechanisms linking disrupted prenatal development and elevated risk of CMD are foetal metabolic adaptations, which may lead to insulin resistance [13], reduced nephron number contributing to higher blood pressure [14], and long-term epigenetic changes affecting metabolic regulation [15]. Therefore, in the present study, we considered an alternative marker of early developmental conditions—facial features. In addition to birth weight, several morphological characteristics have been suggested as markers of developmental (in)stability, including facial fluctuating asymmetry (FA) and facial averageness (AVE), that may reflect early-life environmental quality.

Facial features have been proposed as indicators of developmental stability and resilience to prenatal environmental challenges [16, 17], thus a potential proxy for biological condition [18]. Research suggests that individuals developing under favourable environmental conditions are better able to maintain symmetrical, average morphological traits. In contrast, exposure to disease, poor nutrition, or overall adverse environmental conditions can disrupt developmental processes and increase asymmetry. Therefore, higher facial symmetry and averageness are often interpreted as signals of stable development and better phenotypic quality, whereas increased asymmetry may indicate developmental instability resulting from early-life stressors [17, 19, 20].

Low FA may indicate developmental stability and better health, though evidence is mixed [21]. Stephen *et al.* [22] found that facial shape reflects physiological health (body fat, body mass index, blood pressure) in young adults, but FA and AVE were not assessed. Farrera highlighted the need for integrated models to clarify FA-health links, and that further research is required to confirm FA’s role as a health biomarker [23]. In Klimek *et al*., we previously examined the relationship between facial shape and reproductive history, finding that women with more symmetric faces had an earlier age at first reproduction and, in consequence, a greater number of children. So, the relationship between facial symmetry and number of offspring was not direct and was mediated by the age at first reproduction, although the size of the effect was rather low-to-moderate (*P* = .03, *R*^2^ = 0.16) [24].

Recent studies have found no significant links between FA and CMDs risk factors. Nunes *et al*. observed greater facial disparity in elderly Brazilians with diabetes, but not with hypertension [25]. Tomkinson *et al*. and Penke *et al.* similarly reported no significant associations between facial asymmetry and blood pressure, cholesterol, diabetes, or cardiovascular disease [26, 27].

While some research examined facial traits and cardiometabolic risk, none focused on post-reproductive women. Our study addresses this gap, evaluating the associations between FA, AVE, and cardiometabolic markers in older women—the first to do so exclusively in this population.

## MATERIAL AND METHODS

### Participants

The study sample consisted of 248 non-smoking women aged between 45 and 92 years (Mean = 61.6, SD = 10.75; Table 1). The data were collected between 2012 and 2014 among rural women from several villages located at the Mogielica Human Ecology Study Site in southern Poland [28]. The participants belonged to a rural community characterized by a seasonally changing lifestyle that included high levels of physical activity related to agricultural work. Trained assistants visited each household in the villages to invite women to participate in the study. If a woman agreed to participate, structured questionnaires were administered to collect demographic and lifestyle data, including age, smoking status, and number of years of education as a proxy for socioeconomic status [29]. Additionally, body composition analysis, including body fat percentage, was conducted using bioelectrical impedance analysis, with a Tanita scale BC-545. Waist circumference and blood pressure were also measured. Afterwards, the participants were invited to the local health clinics for the collection of fasting blood samples and facial photographs. Photographs were standardized for facial expression (neutral), distance from the photographer, background, and taken by the same research team member, and all participants reported no history of facial injuries or facial surgeries. The Jagiellonian University Bioethical Committee approved the study protocol (KBET/328/B/2012 and KBET/5B/2010). All participants gave informed written consent.

**Table 1:** Descriptive statistics for participants’ characteristics (*N* = 248).

	Mean	SD	Min.	Median	Max.
FA	0.05	0.02	0.02	0.04	0.12
FA scaled	0.30	0.10	0.00	0.30	1.00
AVE	0.06	0.02	0.03	0.06	0.12
AVE scaled	0.40	0.20	0.00	0.30	1.00
Total cholesterol [mmol/l]	5.62	1.09	2.23	5.68	8.42
HDL [mmol/l]	1.39	0.32	0.74	1.35	2.68
LDL [mmol/l]	3.46	0.95	0.85	3.45	5.84
Triglycerides [mmol/l]	1.43	0.74	0.40	1.29	6.87
Fasting glucose level [mmol/l]	5.49	1.89	3.58	5.16	24.90
Systolic blood pressure [mmHg]	134.80	20.60	90.00	131.00	210.00
Diastolic blood pressure [mmHg]	84.40	11.90	56.00	83.00	137.00
Pulse pressure	50.40	14.30	22.00	48.00	106.00
Age [years]	61.60	10.70	44.80	61.30	92.40
Education [years]	10.30	3.54	0.00	10.00	22.50
Body fat [%]	36.60	6.96	10.70	37.50	54.00

FA scaled, normalized facial fluctuating asymmetry; AVE scaled—normalized facial averageness.

### Facial measurements

For the facial analyses, seven bilateral and two unpaired anthropometric measurement points were digitized on each standardized frontal facial portrait. These points represent the maximum number of bilateral points that were reliably identifiable via facial tissue morphology and/or geometric homology in all participants included in the final sample (fixed landmarks): eyes—endocanthion, exocanthion; nose—Alae origin, Alare; mouth—Cheilion, Christa philtre; jaw line—zygion. Two further unpaired fixed landmarks were placed along or close to the facial midline: Stomion and Gnathion. All landmark operationalizations are described in detail in Windhager *et al*. [30], following Kolar and Salter [31] and Farkas [32]. These landmarks have already proved to be useful for assessing female FA [33]. The Cartesian coordinates of the landmarks were then analysed using geometric morphometrics. Its main advantage is considering the face as a single geometric shape. Procrustes symmetry analysis encompasses three concepts: total asymmetry (TA, found in the complete landmark configuration under consideration by interchanging pairs of landmarks and comparing the original configurations and their relabelled reflections), directional asymmetry (the squared shape distance between the group mean of the original data and the group mean of the mirrored data), and FA i.e. the deviation of within-case asymmetry vectors from their average [34]. Higher scores indicate greater FA. AVE was calculated as the Procrustes distance between the average facial configuration of the sample and the individual facial configuration in tpsSmall 1.34 [35]. Therefore, lower scores reflect higher averageness. Landmarks were digitized in tpsDig2 [35], and asymmetry analyses were conducted in Wolfram Mathematica 12. Both FA and AVE were scaled by normalization—the scaled variable = (value − min(var))/(max(var) − min(var)). Sexual dimorphism in facial shape was not included for both conceptual and methodological reasons. Sexual dimorphism in facial traits emerges mainly during adolescence and is influenced by pubertal hormonal changes, affecting facial appearance and perceived femininity [36, 37]. In contrast, FA and AVE are considered markers of developmental stability, reflecting variation arising from environmental and genetic perturbations in early development [18]. For this reason, we included two facial features: FA and AVE.

### Markers of CMDs risk

CMDs risk markers measured included waist circumference, blood pressure, lipid profile, and glucose. Waist circumference was measured three times and averaged; one participant who declined this measurement was excluded from related models. Blood pressure was measured with an M10-IT automatic sphygmomanometer (Omron, Kyoto, Japan). Pulse pressure was calculated for each participant as the difference between the systolic and DBP. Triglycerides (TG) [mmol/l], HDL-cholesterol [mmol/l], LDL-cholesterol [mmol/l], and glucose level [mmol/l] were analysed by a commercial laboratory using the XS-1000i haematology analyser (Sysmex, Kobe, Japan) and the Pentra 400 analyser (Horiba ABX SAS, Montpellier, France).

Dyslipidaemia, dysglycaemia, and hypertension risks were calculated as dichotomized CMDs risk factors according to the regulations of the Polish Society of Hypertension [38]. Atherogenic dyslipidaemia was defined as TG ≥1.7 mmol/l and HDL-cholesterol <1.3 mmol/l [39], dysglycaemia as elevated fasting plasma glucose ≥5.6 mmol/l [40], hypertension as ≥130/80 mmHg, and pulse pressure as ≥60 mmHg.

MS was defined by a waist circumference ≥88 cm plus at least two of the following: fasting glucose ≥100 mg/dl, non-HDL cholesterol ≥130 mg/dl, or blood pressure ≥130/80 mmHg [38]. Data on medical conditions and medication use were self-reported and were not verified against medical records. Therefore, these data were not included in the calculations of MS. Diagnosis of MS in women followed the Polish Society of Hypertension guidelines, similar to the 1999 WHO definition, except that 2-h post-glucose insulin levels were not measured [38]. Additionally, the 10-year risk of a cardiovascular event (stroke, myocardial infarction, death) was calculated for each individual using the SCORE. The SCORE value, estimated based on sex, smoking status, SBP, and TC, was used to classify risk into three categories: low or moderate, high, and very high. Very high risk was defined differently depending on the age of the participant: for <50 years: ≥7.5%, for 50–69: ≥10%, and for >70 years: ≥15% [41].

### Statistical analysis

We analysed FA and AVE in two separate sets of models. Prior to the main analyses, Spearman correlations were computed to examine bivariate associations between facial traits and all study variables. Firstly, we used hierarchical linear models to test for an association between facial traits (FA or AVE) and each CMDs’ biomarkers. Age, education, and body fat percentage were included as covariates, except in MS models, where body fat was excluded due to its strong correlation with waist circumference. Facial trait measures were added in the second step [42]. Since frequentist statistics do not allow for direct support of the null hypothesis, only fail to reject it, we followed up by computing Bayes Factors (BF_10_) using the BayesFactor package (v 0.9.12-4.7) for R with Liang *et al*.’s [43] mixed *g* priors.

Supplementary hierarchical logistic regressions were conducted using clinically defined CMDs risk thresholds. Two composite outcomes—MS and high SCORE risk—were analysed as dichotomous variables. Age and education were entered in the null model, with facial traits (FA or AVE) added in the second step. Model significance was assessed with chi-squared deviance tests, and Bayes Factors were computed using the BAS package (v1.7.5) in R [44]. The R Markdown for the detailed analyses can be found in the Electronic Supplementary Material.

## RESULTS

### Descriptive statistics

Participants had a mean age of 61.60 ± 10.70 years and 10.30 ± 3.54 years of education. Mean FA was 0.05 ± 0.02, and AVE was 0.06 ± 0.02. Mean TC was 5.62 ± 1.09 mmol/l, LDL 3.46 ± 0.95, HDL 1.39 ± 0.32, and triglycerides 1.43 ± 0.74. Fasting glucose averaged 5.49 ± 1.89 mmol/l (range up to 24.9). Mean systolic/diastolic pressures were 134.80 ± 20.60 mmHg and 84.40 ± 11.90 mmHg, respectively. Pulse pressure averaged 50.40 ± 14.30 mmHg. Body fat averaged 36.6 ± 6.96% (range 10.7%–54.0%). Overall, 35.5% (*n* = 88) were classified as ‘very high risk’ by SCORE, and 55% (*n* = 136) met criteria for MS. Spearman correlations were conducted to examine associations between FA, AVE, and CMDs indicators. As shown in Table 2, neither FA nor AVE was significantly correlated with any of the examined health variables (all |ρ| ≤ 0.11; all *P*s > .05). See Supplementary Tables S1 in Electronic Supplementary Materials for Spearman’s bivariate correlations among all study variables. 

**Table 2:** Spearman correlations between FA, AVE, and cardiometabolic markers.

**Variable**	**FA ρ**	***P***	**AVE ρ**	***P***
Total cholesterol [mmol/l]	−0.02	.74	−0.10	.12
HDL [mmol/l]	−0.09	.16	−0.09	.18
LDL [mmol/l]	−0.03	.66	−0.12	.09
Triglycerides [mmol/l]	0.10	.11	0.04	.49
Fasting glucose level [mmol/l]	0.06	.33	0.05	.45
Systolic blood pressure [mmHg]	0.00	.99	0.05	.46
Diastolic blood pressure [mmHg]	0.05	.44	0.03	.57
Pulse pressure	−0.01	.91	0.04	.53
Age [years]	−0.02	.81	0.04	.50
Education [years]	0.09	.15	0.03	.62
Body fat [%]	0.03	.65	−0.10	.11

FA, facial fluctuating asymmetry; AVE, facial averageness.

### Cardiometabolic disease risk factors and facial asymmetry—linear approach

For TC, the null model was significant (*F*_3,244_ = 3.76, *P* = .011, *R*^2^_adj_ = 0.03), with age as the only significant predictor. Adding FA to the model did not significantly improve the predictive value (*F*_change1,243_ = 0.01, *P* = .913, *R*^2^_change_ = 0.00). Also, adding AVE to the null model did not significantly improve the predictive value (*F*_change1,243_ = 1.77, *P* = .184, *R*^2^_change_ = 0.01). Bayesian analysis gave moderate support for the hypothesis that FA did not improve the predictive value of the model (BF_10_ = 0.274) and anecdotal evidence that AVE did not improve the predictive value of the model (BF_10_ = 0.610).

For HDL cholesterol, the null model was significant (*F*_3,244_ = 7.22, *P* < .001, *R*^2^_adj_ = 0.07), with body fat percentage as the only significant predictor. Adding FA to the model did not significantly improve the predictive value (*F*_change1,243_ = 1.47, *P* = .226, *R*^2^_change_ = 0.01), adding AVE to the null model also did not significantly improve the predictive value (*F*_change1,243_ = 0.27, *P* = .606, *R*^2^_change_ = 0.00). Bayesian analysis provided anecdotal evidence to the hypothesis that FA did not improve the predictive value of the model (BF_10_ = 0.493) and moderate support for AVE not improving the predictive value of the model (BF_10_ = 0.283).

For LDL cholesterol, the null model was significant (*F*_3,244_ = 4.02, *P* = .008, *R*^2^_adj_ = 0.04), with age as the only significant predictor. Adding FA to the model did not significantly improve the predictive value (*F*_change1,243_ = 0.00, *P* = .946, *R*^2^_change_ = 0.00), adding AVE to the null model also did not significantly improve the predictive value (*F*_change1,243_ = 1.78, *P* = .183, *R*^2^_change_ = 0.01). Bayesian analysis gave moderate support for the hypothesis that FA did not improve the predictive value of the model (BF_10_ = 0.271) and anecdotal evidence that AVE did not improve the predictive value of the model (BF_10_ = 0.609).

For triglycerides, the null model was significant (*F*_3,244_ = 3.24, *P* = .023, *R*^2^_adj_ = 0.03), with body fat percentage as the only significant predictor. Adding FA to the model did not significantly improve the predictive value (*F*_change1,243_ = 1.28, *P* = .258, *R*^2^_change_ = 0.01), adding AVE to the null model also did not significantly improve the predictive value (*F*_change1,243_ = 0.00, *P* = .986, *R*^2^_change_ = 0.00). Bayesian analysis provided anecdotal evidence for the hypothesis that FA did not improve the predictive value of the model (BF_10_ = 0.495) and moderate support for AVE not improving the predictive value of the model (BF_10_ = 0.276).

For glucose, the null model was significant (*F*_3,244_ = 6.74, *P* < .001, *R*^2^_adj_ = 0.07), with age as the only significant predictor. Adding FA to the model did not significantly improve the predictive value (*F*_change1,243_ = 0.03, *P* = .864, *R*^2^_change_ = 0.00), adding AVE to the null model also did not significantly improve the predictive value (*F*_change1,243_ = 0.01, *P* = .941, *R*^2^_change_ = 0.00). Bayesian analysis gave moderate support for the hypotheses that both FA and AVE did not improve the predictive value of the model (BF_10_ = 0.256) and (BF_10_ = 0.254), respectively.

For SBP, the null model was significant (*F*_3,244_ = 7.79, *P* < .001, *R*^2^_adj_ = 0.08), with age as the only significant predictor. Adding FA to the model did not significantly improve the predictive value (*F*_change1,243_ = 0.15, *P* = .696, *R*^2^_change_ = 0.00), adding AVE to the null model also did not significantly improve the predictive value (*F*_change1,243_ = 0.52, *P* = .470, *R*^2^_change_ = 0.00). Bayesian analysis gave moderate support for the hypotheses that both FA and AVE did not improve the predictive value of the model (BF_10_ = 0.265) and (BF_10_ = 0.314), respectively.

For DBP, the null model was not significant (*F*_3,244_ = 0.97, *P* = .406, *R*^2^_adj_ = 0.00). Adding FA to the model did not significantly improve the predictive value (*F*_change1,243_ = 0.74, *P* = .568, *R*^2^_change_ = 0.00), adding AVE to the null model also did not significantly improve the predictive value (*F*_change1,243_ = 0.35, *P* = .554, *R*^2^_change_ = 0.00). Bayesian analysis gave moderate support for the hypothesis that FA did not improve the predictive value of the model (BF_10_ = 0.299) and anecdotal evidence that AVE did not improve the predictive value of the model (BF_10_ = 0.345).

For pulse pressure, the null model was significant (*F*_3,244_ = 23.45, *P* < .001, *R*^2^_adj_ = 0.21), with age as the only significant predictor. Adding FA to the model did not significantly improve the predictive value (*F*_change1,243_ = 0.19, *P* = .664, *R*^2^_change_ = 0.00), adding AVE to the null model also did not significantly improve the predictive value (*F*_change1,243_ = 0.33, *P* = .569, *R*^2^_change_ = 0.00). Bayesian analysis gave moderate support for the hypotheses that both FA and AVE did not improve the predictive value of the model (BF_10_ = 0.204) and (BF_10_ = 0.218), respectively.

For full results and detailed model estimates, see Table 3 for FA and Table 4 for AVE.

**Table 3:** Linear regression results: coefficients [95% CI] for the overall models predicting cardiometabolic markers from age, body fat %, education, and facial fluctuating asymmetry (*N* = 248).

	**Total cholesterol** [mmol/l]	**HDL** [mmol/l]	**LDL** [mmol/l]	**Triglycerides** [mmol/l]	**Glucose** [mmol/l]	**Systolic** **blood pressure** [mmHg]	**Diastolic** **blood** **pressure** [mmHg]	**Pulse** **pressure**
**Intercept**	7.35 [5.83, 8.88]	1.87 [1.43, 2.30]	4.87 [3.53, 6.20]	1.02 [−0.01, 2.06]	2.64 [0.03, 5.25]	102.96 [74.80, 131.12]	89.89 [72.85, 106.93]	13.07 [−4.92, 31.06]
	*P* < .001	*P* < .001	*P* < .001	*P* = .053	*P* = .048	*P* < .001	*P* < .001	*P* = .154
**Age** **[years]**	−0.02 [−0.03, −0.00]	−0.00 [−0.01, 0.00]	−0.01 [−0.03, −0.00]	−0.00 [−0.01, 0.01]	0.03 [0.01, 0.06]	0.60 [0.30, 0.89]	−0.04 [−0.21, 0.14]	0.63 [0.45, 0.82]
	*P* = .024	*P* = .437	*P* = .036	*P* = .582	*P* = .016	*P* < .001	*P* = .692	*P* < .001
**Education [years]**	−0.00 [−0.05, 0.04]	0.01 [−0.01, 0.02]	0.00 [−0.04, 0.05]	−0.02 [−0.05, 0.01]	−0.04 [−0.12, 0.05]	0.14 [−0.76, 1.03]	0.14 [−0.41, 0.68]	0.00 [−0.57, 0.57]
	*P* = .874	*P* = .302	*P* = .857	*P* = .283	*P* = .396	*P* = .761	*P* = .622	*P* = .992
**Body fat [%]**	−0.01 [−0.03, 0.00]	−0.01 [−0.02, −0.01]	−0.01 [−0.03, 0.00]	0.02 [0.01, 0.03]	0.03 [−0.00, 0.06]	−0.20 [−0.57, 0.16]	−0.14 [−0.36, 0.08]	−0.07 [−0.30, 0.16]
	*P* = .136	*P* < .001	*P* = .112	*P* = .007	*P* = .081	*P* = .267	*P* = .224	*P* = .558
**FA scaled**	−0.05 [−0.95, 0.85]	−0.16 [−0.42, 0.01]	−0.03 [−0.82, 0.76]	0.35 [−0.26, 0.97]	0.13 [−1.41, 1.68]	3.31 [−13.35, 19.97]	0.96 [−9.12, 11.05]	2.35 [−8.30, 12.99]
	*P* = .913	*P* = .226	*P* = .946	*P* = .258	*P* = .864	*P* = .696	*P* = .851	*P* = .664
**Model sig.**	*P* = .026	*P* < .001	*P* = .019	*P* = .029	*P* < .001	*P* < .001	*P* = .568	*P* < .001
***R*** ^**2**^	0.044	0.087	0.047	0.043	0.077	0.088	0.012	0.224

FA scaled, normalized facial asymmetry.

**Table 4:** Linear regression results: coefficients [95% CI] for the overall models predicting cardiometabolic markers from age, body fat %, education, and facial averageness (*N* = 248).

	**Total cholesterol** [mmol/l]	**HDL** [mmol/l]	**LDL** [mmol/l]	**Triglycerides** [mmol/l]	**Glucose** [mmol/l]	**Systolic** **blood pressure** [mmHg]	**Diastolic** **blood** **pressure** [mmHg]	**Pulse** **pressure**
**Intercept**	7.52 [5.99, 9.04]	1.85 [1.41, 2.29]	5.01 [3.68, 6.35]	1.11 [0.06, 2.16]	2.66 [0.03, 5.28]	101.97 [73.69, 130.25]	89.24 [72.12, 106.36]	12.73 [−5.35, 30.81]
	*P* < .001	*P* < .001	*P* < .001	*P* = .038	*P* = .048	*P* < .001	*P* < .001	*P* = .167
**Age** **[years]**	−0.02 [−0.03, −0.00]	−0.00 [−0.01, 0.00]	−0.02 [−0.03, −0.00]	−0.00 [−0.01, 0.01]	0.03 0.01, 0.06]	0.60 [0.30, 0.89]	−0.04 [−0.22, 0.14]	0.63 [0.44, 0.82]
	*P* = .023	*P* = .454	*P* = .035	*P* = .564	*P* = .016	*P* < .001	*P* = .690	*P* < .001
**Education [years]**	−0.01 [−0.05, 0.04]	0.01 [−0.01, 0.02]	0.00 [−0.04, 0.04]	−0.02 [−0.05, 0.02]	−0.04 −0.12, 0.05]	0.17 [−0.73, 1.06]	0.15 [−0.39, 0.69]	0.02 [−0.56, 0.59]
	*P* = .800	*P* = .333	*P* = .932	*P* = .294	*P* = .402	*P* = .717	*P* = .591	*P* = .954
**Body fat [%]**	−0.01 [−0.03, 0.00]	−0.01 [−0.02, −0.01]	−0.01 [−0.03, 0.00]	0.02 [0.01, 0.03]	0.03 [−0.00, 0.06]	−0.20 [−0.56, 0.16]	−0.14 [−0.35, 0.08]	−0.07 [−0.30, 0.16]
	*P* = .144	*P* < .001	*P* = .120	*P* = .005	*P* = .077	*P* = .269	*P* = .221	*P* = .570
**AVE scaled**	−0.48 [−1.20, 0.23]	−0.05 [−0.26, 0.15]	−0.43 [−1.05, 0.20]	−0.00 −0.50, 0.49]	0.05 [−1.19, 1.28]	4.88 [−8.40, 18.16]	2.42 [−5.62, 10.46]	2.46 [−6.03, 10.95]
	*P* = .184	*P* = .606	*P* = .183	*P* = .986	*P* = .941	*P* = .470	*P* = .554	*P* = .569
**Model sig.**	*P* = .012	*P* < .001	*P* = .009	*P* = .049	*P* < .001	*P* < .001	*P* = .516	*P* < .001
***R*** ^**2**^	0.051	0.083	0.054	0.038	0.077	0.089	0.013	0.225

AVE scaled, normalized facial averageness.

### Composite and dichotomized measures

The base model with age, years of education, and body fat percentage significantly predicted SCORE (*P* < .001). Separately, adding FA (*P*_change_ = .200) or AVE (*P*_change_ = .785; Table 4) to this model did not significantly improve its predictive value. Bayesian hierarchical logistic regression provided moderate evidence that both FA (BF_10_ = 0.227) and AVE (BF_10_ = 0.104) did not improve the null model.

The base model containing age and years of education significantly predicted MS (*P* = .001). Separately adding FA (*P*_change_ = .266) and AVE (*P*_change_ = .623; Table 3) to the model did not significantly improve its predictive value. Bayesian hierarchical logistic regression provided moderate evidence that neither FA (BF10 = 0.198) nor AVE (BF10 = 0.121) improved the predictive value of the model.

For full results and detailed model estimates, see Table 5.

**Table 5:** Logistic regression of metabolic syndrome (*N* = 247), SCORE (*N* = 248), and facial fluctuating asymmetry and facial averageness, adjusted for age, education, and body fat% for the SCORE scale.

	**Metabolic syndrome**	**SCORE**
**Intercept**	−0.04 [−2.68, 2.59]	−19.96 [−27.50, −13.66]
	*P* = .975	*P* < .001
**FA scaled**	1.24 [−0.46, 3.02]	1.82 [−0.97, 4.67]
	*P* = .160	*P* = 0.201
**Age [years]**	0.00 [−0.03, 0.03]	0.32 [0.24, 0.42]
	*P* = .844	*P* < .001
**Education [years]**	−0.03 [−0.12, 0.06]	−0.03 [−0.22, 0.15]
	*P* = 0.523	*P* = .712
**Body fat [%]**	N/A	−0.04 [−0.11, 0.02]
	N/A	*P* = .216
**Intercept**	0.44 [−2.18, 3.07]	−19.95 [−27.67, −13.48]
	*P* = .742	*P* < .001
**AVE scaled**	−0.21 [−1.56, 1.14]	−0.32 [−2.60, 2.01]
	*P* = .759	*P* = .784
**Age [years]**	0.00 [−0.03, 0.03]	0.32 [0.24, 0.43]
	*P* = .877	*P* < .001
**Education [years]**	−0.03 [−0.12, 0.06]	−0.01 [−0.19, 0.17]
	*P* = .518	*P* = .913
**Body fat [%]**	N/A	−0.03 [−0.10, 0.03]
	N/A	*P* = .304

FA scaled, normalized facial fluctuating asymmetry; AVE scaled, facial averageness normalized.

In models including dichotomized dependent variables other than SCORE and MS (dyslipidaemia, high pulse pressure, hypertension, dysglycaemia), we did not observe any significant relationships. Detailed results are presented in the Electronic Supplementary Materials (Supplementary Tables S2 and S3).

## DISCUSSION

We found no evidence of an association between facial FA or averageness and cardiometabolic risk indicators when controlling for age, body fat percentage, and years of education. The importance of developmental conditions for later health is well established [45]. Accordingly, traits proposed as reliable biomarkers of developmental conditions are expected to predict disease risks in adulthood. This study offers a comprehensive assessment of the relationships between facial FA and averageness and the risk of CMDs in post-reproductive age women. We provide evidence against the association between FA and all measured CMDs risk factors. Our findings are consistent with those of Tomkinson *et al*. and Penke *et al*., both of whom found no association between FA and the lipid profile or blood pressure [26, 27]. Similarly, Nunes *et al*. reported no difference in FA between individuals with and without hypertension [25]. Our null findings also suggest that AVE may not be a reliable indicator of cardiometabolic health. Rhodes *et al*. found that in an adult sample, averageness and symmetry increased perceived health; however, medical records revealed only limited associations between distinctiveness and actual health, and no associations for asymmetry [46]. However, prior studies consistently highlighted the confounding role of adiposity and BMI, rather than facial structure alone, in predicting metabolic risk [47].

While our findings broadly align with those of previous studies, some methodological differences deserve consideration, as other authors focus on slightly different aspects of facial morphology. In our study, facial measures were derived from seven bilateral anthropometric measurement points and two unpaired ones. Nunes *et al*. [25] used four bilateral and one midline point, while Penke *et al*. [27] applied seven bilateral landmarks (five identical to ours), one midline. Tomkinson *et al*. [26] used a different approach, originally employed by Grammer and Thornhill [17], in which only horizontal distances from the facial midline were measured, ignoring vertical asymmetry altogether. The fact that multiple methods for quantifying facial FA have failed to identify a consistent link between facial asymmetry and markers of cardiovascular health in adulthood further strengthens the case for a lack of such an association, at least in studies based on ‘WEIRD’ (Western, Educated, Industrialized, Rich, Democratic) populations [48].

Our study focuses exclusively on women of postreproductive age. Nunes *et al*. [25] included elderly participants of both genders, similar to Penke *et al*., whose sample consisted of individuals aged 79 to 83. In contrast, Stephen *et al*. [22] and Tomkinson *et al*. [26] included much younger participants, aged 18 to 24 years. However, analysing disease risk in young adults may not capture long-term negative health effects, which are more likely to manifest later in life. As reported in earlier studies, adult facial symmetry reflects the combined influence of genetic and environmental factors, indicating the quality of prenatal and postnatal development [49, 50]. According to previous findings, ageing of soft tissue also increases FA [51], making it important to include participants of varying ages when studying face shape and health and to disentangle these effects in future studies.

Although facial features have been proposed as potential markers of early developmental conditions, their reliability as a biomarker for CMDs risk in adult life remains questionable [23]. This highlights the need for a more comprehensive approach to both health (and its various dimensions) and its biomarkers in future research.

### Strengths, limitations, and future directions

The main strength of the study is the inclusion of a wide array of CMDs risk factors, namely blood pressure, lipid profile, and glucose levels, as well as composite markers of CMDs risk such as MS and the Systematic Coronary Risk Evaluation tool. Moreover, our analyses were based on a homogeneous sample of non-smokers living in a comparable environment (villages in southern Poland), resulting in reduced variation in living conditions that could affect health in older age. Additionally, the results are based on a transparent and robust data analysis process, starting with advanced measurements of FA and AVE using geometric morphometrics and ending with a statistical approach based on Bayesian analyses to assess the strength of evidence for the null hypotheses.

Some limitations of the study should be taken into account. First, our analyses were based on a single blood sample and blood pressure measurement. Second, facial measurements in health-related research are predominantly conducted in populations of European descent (except for the study by Stephen *et al*. [22]). This study population originates from a WEIRD society (Western, Educated, Industrialized, Rich, and Democratic). However, participants primarily came from a physically working community with socioeconomic patterns that differ from highly urbanized or highly educated subgroups typically associated with WEIRD samples. Conceptually speaking, there is no reason why the results should not generalize to non-WEIRD populations. However, there might be populations facing harsher conditions, potentially increasing variation, especially towards more stressful early life environments and greater facial variation, so that subtle effects may become significant. Nevertheless, we do not have empirical evidence to generalize our findings to non-WEIRD populations directly. Therefore, replication in populations with different cultural and socioeconomic backgrounds is desirable. Expanding research to include African or Asian populations is essential to address existing disparities and to account for the increasing prevalence of CMDs in these groups [52]. Additionally, the risk of CVD is strongly influenced by lifestyle factors in adult life. In younger adults, it was found that facial asymmetry—measured as overall asymmetry following Grammer and Thornhill [17]—was independent of age. Thus, the effects of early conditions may not be detectable when measuring adult populations, especially at older ages. Lastly, as the responses in our study were collected from post-menopausal participants only, we did not have the possibility to collect direct indicators of the harshness of the early life conditions, e.g. birth size.

Recent work has questioned the tendency to interpret facial characteristics primarily as cues to immunocompetence, arguing that such accounts may be overly narrow and that lifestyle-related influences on facial appearance should also be considered [53, 54].

## CONCLUSION AND IMPLICATIONS

We found moderate evidence that there are no associations between facial FA or AVE—putative markers of the developmental environment—and risk factors for CMDs among women of post-reproductive age. While facial traits may be relevant in social perception, they are unlikely to serve as biomarkers of increased CMDs risk in older women.

## Supplementary Material

Supplementary_Files_eoag014

## Data Availability

Data supporting the findings of this study are available upon reasonable request from the corresponding author. The data are not publicly available due to privacy and ethical restrictions, as they contain information that could compromise the anonymity of research participants.
