# Supplementary material for: The evaluation of facial features as markers of cardiometabolic health in women of post-reproductive age
Source: Evol Med Public Health. 2026 Jul 2;14(1):eoag014. doi: 10.1093/emph/eoag014 (PMC13387495; doi:10.1093/emph/eoag014)
Supplement: Supplementary_Files_eoag014 [file supplementary_files_eoag014.docx]

**Table S1.** Spearman’s bivariate (zero-order) correlations among all study variables.

|  | FA | AVE | Total Cholesterol [mmol/L] | HDL [mmol/L] | LDL [mmol/L] | Triglycerides [mmol/L] | Fasting  glucose level [mmol/L] | Systolic Blood Pressure [mmHg] | Diastolic Blood Pressure [mmHg] | Pulse Pressure | Age (years) | Education (years) | Body Fat % |
| --- | --- | --- | --- | --- | --- | --- | --- | --- | --- | --- | --- | --- | --- |
| FA | 0.02 | 0.26*** | -0.01 | -0.09 | -0.01 | 0.09 | 0.01 | 0.01 | 0.01 | 0.01 | -0.03 | 0.03 | 0.09 |
| AVE | 0.26*** | 0.07 | -0.10 | -0.05 | -0.10 | 0.01 | 0.03 | 0.06 | 0.03 | 0.06 | 0.06 | -0.09 | 0.05 |
| Total Cholesterol [mmol/L] | -0.01 | -0.10 | 5.44 | 0.31*** | 0.92*** | 0.19** | -0.09 | -0.02 | 0.08 | -0.10 | -0.19** | 0.11 | -0.12 |
| HDL [mmol/L] | -0.09 | -0.05 | 0.31*** | 1.05 | 0.17** | -0.48*** | -0.15* | -0.05 | 0.02 | -0.09 | -0.14* | 0.14* | -0.26*** |
| LDL [mmol/L] | -0.01 | -0.10 | 0.92*** | 0.17** | 5.47 | 0.04 | -0.08 | -0.02 | 0.07 | -0.08 | -0.19** | 0.13* | -0.13* |
| Triglycerides [mmol/L] | 0.09 | 0.01 | 0.19** | -0.48*** | 0.04 | 0.57 | 0.10 | 0.06 | 0.05 | 0.04 | 0.03 | -0.08 | 0.19** |
| Fasting glucose level [mmol/L] | 0.01 | 0.03 | -0.09 | -0.15* | -0.08 | 0.10 | 5.53 | 0.08 | 0.02 | 0.10 | 0.25*** | -0.20** | 0.15* |
| Systolic Blood Pressure [mmHg] | 0.01 | 0.06 | -0.02 | -0.05 | -0.02 | 0.06 | 0.08 | 114.00 | 0.74*** | 0.82*** | 0.29*** | -0.16* | -0.03 |
| Diastolic Blood Pressure [mmHg] | 0.01 | 0.03 | 0.08 | 0.02 | 0.07 | 0.05 | 0.02 | 0.74*** | 75.00 | 0.23*** | -0.07 | 0.07 | -0.09 |
| Pulse Pressure | 0.01 | 0.06 | -0.10 | -0.09 | -0.08 | 0.04 | 0.10 | 0.82*** | 0.23*** | 41.00 | 0.47*** | -0.29*** | 0.03 |
| Age (years) | -0.03 | 0.06 | -0.19** | -0.14* | -0.19** | 0.03 | 0.25*** | 0.29*** | -0.07 | 0.47*** | 73.57 | -0.62*** | 0.14* |
| Education (years) | 0.03 | -0.09 | 0.11 | 0.14* | 0.13* | -0.08 | -0.20** | -0.16* | 0.07 | -0.29*** | -0.62*** | 12.00 | -0.12 |
| Body Fat % | 0.09 | 0.05 | -0.12 | -0.26*** | -0.13* | 0.19** | 0.15* | -0.03 | -0.09 | 0.03 | 0.14* | -0.12 | 29.70 |

*** p < 0.001; ** p < 0.01; * p < 0.05

|  | **Dyslipidemia** | **Hypertension** | **Dysglicemia** | **Pulse Press** |
| --- | --- | --- | --- | --- |
| **Intercept** | -0.34 [-3.23, 2.55] | 0.37 [-2.80, 3.51] | -7.33 [-11.17, -3.70] | -5.84 [-9.68, -2.15] |
|  | p = .819 | p = .817 | p < .001 | p = .002 |
| **FA scaled^1^** | 0.74 [-0.96, 2.48] | 0.41 [-1.43, 2.34] | 0.51 [-1.52, 2.50] | 0.69 [-1.45, 2.75] |
|  | p = .396 | p = .667 | p = .614 | p = .517 |
| **Age (years)** | -0.01 [-0.04, 0.02] | 0.02 [-0.02, 0.05] | 0.06 [0.02, 0.09] | 0.08 [0.04, 0.12] |
|  | p = .465 | p = .361 | p = .003 | p < .001 |
| **Education (years)** | -0.07 [-0.16, 0.03] | -0.00 [-0.10, 0.10] | 0.00 [-0.11, 0.12] | -0.03 [-0.15, 0.09] |
|  | p = .165 | p = .938 | p = .959 | p = .638 |
| **Body Fat %** | 0.04 [0.00, 0.08] | -0.01 [-0.06, 0.03] | 0.06 [0.02, 0.12] | -0.01 [-0.06, 0.04] |
|  | p = .045 | p = .526 | p = .010 | p = .666 |

**Table S2.** Logistic regression results: coefficients [95% CI] and significance of **facial asymmetry** controlling for age, education, and body fat % in predicting dysglycemia, hypertension, dyslipidemia, and pulse pressure (N = 248)

^1^FA scaled – normalized facial asymmetry

The model predicting dysglycemia from age, years of education, and body fat % was significant (χ^2^(3,244)=24.81, p<.001). Adding FA in the second step did not significantly improve the predictive power of the model (χ^2^(1,243)=0.25, p=.616). Bayesian hierarchical logistic regression provided moderate evidence that adding FA in the second step did not improve the model (BF_10_=.120).

The model predicting hypertension from age, years of education, and body fat % was not significant (χ^2^(3,244)=1.62, p=.655, AIC=303.38). Adding FA in the second step did not significantly improve the predictive power of the model (χ^2^(1,243)=0.19, p=.665). Bayesian hierarchical logistic regression provided moderate evidence that adding FA in the second step did not improve the model (BF_10_=.120).

The model predicting dyslipidemia from age, years of education, and body fat % was not significant (χ^2^(3,244)=6.85, p=.077). Adding FA in the second step did not significantly improve the predictive power of the model (χ^2^(1,243)=0.73, p=.394). Bayesian hierarchical logistic regression provided moderate evidence that adding FA in the second step did not improve the model (BF_10_=.156).

The model predicting pulse pressure from age, years of education, and body fat % was significant (χ^2^(3,244)=33.90, p<.001). Adding FA in the second step did not significantly improve the predictive power of the model (χ^2^(1,243)=0.41, p=.519). Bayesian hierarchical logistic regression provided moderate evidence that adding FA in the second step did not improve the model (BF_10_=.128).

**Table S3.** Logistic regression results: coefficients [95% CI] and significance of **facial averageness** controlling for age, education, and body fat % in predicting dysglycemia, hypertension, dyslipidemia, and pulse pressure (N = 248)

|  | **Dyslipidemia** | **Hypertension** | **Dysglicemia** | **Pulse Press** |
| --- | --- | --- | --- | --- |
| **Intercept** | -0.34 [-3.25, 2.56] | 3.38 [-2.23, 9.39] | -7.01 [-10.87, -3.34] | -5.54 [-9.39, -1.81] |
|  | p = .816 | p = 0.25 | p < .001 | p = .004 |
| **AVE scaled^1^** | 0.50 [-0.86, 1.87] | -0.03 [-1.51, 1.48] | -0.75 [-2.45, 0.88] | -0.45 [-2.20, 1.23] |
|  | p = .474 | p = 0.97 | p = .377 | p = .607 |
| **Age (years)** | -0.01 [-0.04, 0.02] | 0.02 [-0.02, 0.05] | 0.06 [0.02, 0.09] | 0.08 [0.04, 0.12] |
|  | p = .452 | p = 0.37 | p = .003 | p <.001 |
| **Education (years)** | -0.06 [-0.16, 0.03] | -0.27 [-0.75, 0.16] | 0.00 [-0.12, 0.11] | -0.03 [-0.15, 0.09] |
|  | p = .186 | p = 0.24 | p = .977 | p = .638 |
| **Body Fat %** | 0.04 [0.00, 0.08] | -0.09 [-0.24, 0.04] | 0.07 [0.02, 0.12] | -0.01 [-0.05, 0.37] |
|  | p = .039 | p = 0.20 | p = .008 | p = .723 |

^1^AVE scaled - normalized facial averageness

The model predicting dysglycemia from age, years of education, and body fat % was significant (χ^2^(3,244)=24.81, p<.001). Adding averageness in the second step did not significantly improve the predictive power of the model (χ^2^(1,243)=0.80, p=.373). Bayesian hierarchical logistic regression provided moderate evidence that adding averageness in the second step did not improve the model (BF_10_=.157).

The model predicting hypertension from age, years of education, and body fat % was not significant (χ^2^(3,244)=1.62, p=.655). Adding averageness in the second step did not significantly improve the predictive power of the model (χ^2^(1,243)=0.00, p=.973). Bayesian hierarchical logistic regression provided moderate evidence that adding averageness in the second step did not improve the model (BF_10_=.110).

The model predicting dyslipidemia from age, years of education, and body fat % was not significant (χ^2^(3,244)=6.85, p=.077). Adding averageness in the second step did not significantly improve the predictive power of the model (χ^2^(1,243)=0.52, p=.473). Bayesian hierarchical logistic regression provided moderate evidence that adding averageness in the second step did not improve the model (BF_10_=.140).

The model predicting pulse pressure from age, years of education, and body fat % was significant (χ^2^(3,244)=33.90, p<.001). Adding averageness in the second step did not significantly improve the predictive power of the model (χ^2^(1,243)=0.27, p=.605). Bayesian hierarchical logistic regression provided moderate evidence that adding averageness in the second step did not improve the model (BF_10_=.119).
